# How COVID-19 impacted cultural consumption: an explorative analysis of Gen Z’s digital museum experiences

**DOI:** 10.1007/s43039-023-00071-6

**Published:** 2023-03-30

**Authors:** Elena Bonel, Mauro Capestro, Eleonora Di Maria

**Affiliations:** Department of Economics and Management “Marco Fanno”, University of Padova, Via del Santo, 33, 35123 Padua, Italy

**Keywords:** COVID-19, Cultural consumption, Digital experience, Museums, Website

## Abstract

Since the outbreak of the COVID-19 pandemic, arts and culture have experienced a paradoxical situation. The sector has been one of the most negatively affected due to both mobility restrictions and social distancing; yet for the same reasons, demand for online cultural experiences has intensified, and digital access has become more critical than ever. While extant research has focused mainly on the offering side of such changes and developments, this paper investigates how the pandemic impacted on cultural consumption online, and particularly for museum products. The research takes on a demand perspective, an often neglected one when analyzing digitalization and innovation in museums’ offering. Through a direct survey approach, the paper addresses a gap in research on younger subjects, the Generation Z, typically a non-demand segment. It specifically investigates their reactions to digital museum experiences, a realm that research based on museum data has pointed out as a probable route to their involvement. The research is based on two surveys on cultural consumption of a sample of Italian university students of over 1000 respondents, before and during lockdown. Results show that during COVID-19 not only has online cultural consumption increased, but so has the quality of the associated experience, where online museum visits are concerned. Results indicate that learning processes occurred during the pandemic, suggesting potential changes in cultural consumption mediated by digital technologies, with implications for multi-channel strategies of museums in the post-pandemic scenario.

## Introduction

The COVID-19 pandemic has deeply impacted on consumers’ activities both in terms of purchasing processes, and product consumption. Specifically, during lockdown customers have experienced limitations in accessing distribution for information gathering, product selection and purchase, as well as in service use (Sheth, 2020). This scenario can be particularly relevant to analyze when considering cultural consumption that is included in the experience economy (Pallud & Straub, 2014; Pine & Gilmore, 1999). Due to COVID-19 lockdown, the possibility for customers to physically visit museums was particularly reduced, opening questions on whether consumer behavior related to cultural fruition would have been affected or modified over time and on the role of digital technologies in this path. Scholars have dedicated lots of attention to the evolution of museum organizations towards more innovative product-service offering systems, revenue increase and audience development (Cerquetti, 2016; Lindqvist, 2012). Research of this stream has also addressed, as recently spurred by COVID-19, museum digitalization and its effects on museums’ attractiveness (Ryder et al., 2021; Palumbo, 2022; Raimo et al., 2021), as well its potential limits (Black, 2012; Gheorghilaș et al., 2017). However, most studies are based on secondary data, and more customer-centric analysis has received instead comparably less attention. This is particularly true for specific categories of museums’ audience such as the Generation Z group (Gen Z) (Batat, 2020; Tranta et al., 2021). Gen Z may well be different from traditional cultural audiences, particularly in terms of digital habits (Batat, 2020; Kushwaha, 2021; Ryder et al., 2021; Sabaitytė et al., 2017).

Nevertheless, directly investigating the consumer’s perspective could better provide an understanding of how consumers approach the museums’ proposals in times of crisis. This is particularly significant, within a broader scenario of multifaced and not always fully developed online presence of museums. Indeed, a specific focus of research on non-visitors and even more specifically on Generation Z, who are recognized in literature as a typical non-visitors group much to museums and researchers’ concern, has been called for (Batat, 2020; Hughes & Moscardo, 2019; Kluge-Pinsker & Stauffer, 2021; Tranta et al., 2021).

Digital technologies may reinforce the relationship between the customer and the organization, be it a firm or a museum (Palumbo, 2022; Ryder et al., 2021) as they allow overcoming the limitation of distance (for interaction and purchasing processes as well as time management optimization), further exacerbated by the COVID-19 pandemic. Cultural consumption activities are peculiar, and they may be affected by the conditions on the customer’s side. In fact, cultural consumption entails several layers of activation in the consumer, and different consumer backgrounds can lead to different activations, to the extent that some groups might not being able to recognize as art or heritage what is being proposed by cultural organizations; this is particularly relevant for prototypically hard-to-get and yet very much sought after consumer groups such as young people, and particularly, for Generation Z, a group displaying novel ways of conceiving the concepts of art and heritage, and of interacting with culture (Batat, 2020; Hughes & Moscardo, 2019). Thus, Generation Z appear to be particularly resistant to becoming museum visitors, and literature has suggested that a digital approach on the part of museums could be opening the way for reaching out to this group (Manna & Palumbo, 2018; Batat, 2020; Tranta 2021).

Cultural activities can also be impacted by the level of digitalization characterizing the offering. In this sense, the pandemic has provided researchers with potential for testing this tenet (Arnaboldi & Diaz Lema, 2021a, b), both because of increasing levels in museum digitalization (Palumbo, 2022; Raimo et al., 2021; Ryder et al., 2021), and of an increase in online presence across most population categories.

Studies have emphasized the potential for museums in the digitalization process *vis-à-vis* the relationship with their audience (Aizpuru et al., 2019; Hume, 2011). A wide set of technologies can be used to create cultural products’ experiences, enhancing the education and/or entertainment dimensions both in presence and not (Bolin, 2016; Giannini & Bowen, 2019; Pei & Liu, 2019; Rizvic et al., 2020), also supporting consumer data collection and management for audience development (Kabassi, 2020; Mulholland et al., 2016; Pei & Liu, 2019; Sylaiou et al., 2010). The web and social media reduced the distance between customer and offering, in terms of information accessibility as well as rich online contents (Appel et al., 2020; Lin et al., 2012). More recent technologies going under the Industry 4.0 umbrella–such as virtual and augmented reality (VR and AR)–further enhance the physical/digital spheres (Alinam et al., 2020; Marques & Costello, 2018; Vavoula et al., 2009), increasing the customers’ opportunity to more deeply understand the product and its use in the context creating new experiences (Radermecker, 2021).

This process was still meeting much resistance in many institutions, when COVID19 disrupted the scene (Raimo et al., 2021). The relationship of museum institutions with the virtualization of heritage has been evolving, both within the museum, and online. Research also shows that not all museums are aligned with this trend (Agostino and Arnaboldi, 2017; Maida, 2021).

It is extant that the COVID-19 pandemic created a reaction in museums, accelerating digitalization and internet presence (NEMO, 2021). Further attention is related to the consequences of the pandemic on the opportunities and threats of cultural consumption in a digital setting (Palumbo, 2022; Raimo et al., 2021). A growing body of research, and a growing data collection process, has focused on the supply side transformation (Agostino et al., 2020, 2021; Antara & Sen, 2020; Feldman, 2020; Kahn, 2020; Potts, 2020; Samaroudi et al., 2020). However, while research has considered the transformation happening on the supply side, further attention should be devoted to consider the demand side, too (Kluge-Pinsker & Stauffer, 2021; Potts, 2020; Tranta et al., 2021). This need is also endorsed by the now recognized importance of considering museums as places for “enjoyment”, giving more weight to demand issues including novel fruition ways and cocreation (ICOM, 2022).

Along the lines of some new research appearing (Radermecker, 2021, Solima & Cicerchia, 2020; Cicerchia & Solima, 2021; Tranta et al., 2021) this paper investigates how the pandemic has affected cultural and museum consumption online, with specific attention on the Gen Z, a consumer group that is typically a non-visitor one, and one that is both sought after in the industry, and recognized as under-researched in literature, as detailed above. Based on a two-steps survey of museum consumers, conducted in 2020 at the start and at the end of the first lockdown in Italy, the study explores how the lockdown and the linked higher time spent online by individuals have influenced a Gen Z consumer sample’s digital experiences with respect to online museum visiting. Results carry implications for museum operators, as well as suggestions for further research into if and how Z Generation, and consumers at large, have been evolving their cultural consumption patterns as related to the pandemic, and how permanently.

## Theoretical background

### Cultural consumption and digitalization

When cultural consumption is specifically considered, “the consumption process remains experiential, characterized by different levels of proximity and interactions with the good and service.” (Radermecker, 2021, p. 3)**.** The trend to enhance consumer involvement, and the attention to consumer experience and enjoyment stemmed from financial issues, pushing museums to increase visitor numbers (Cerquetti, 2016), as well as from a new concept of the role of museums not anymore only as custodians of heritage goods, but as innovators and cultural service providers too (Van Aalst & Boogaarts, 2002; Katz-Gerro, 2004; Black, 2012; Camarero, Garrido and Vicente, 2015; Mulholland et al., 2016; Agostino et al., 2021; ICOM International, 2022). This evolution may be particularly interesting in involving new generations of cultural audience such as the Gen Z, who have distinctive characteristics and requirements with respect to other audiences, in terms of values, attitude towards digitalization and the like (Batat, 2020; Hughes & Moscardo, 2019; Manna & Palumbo, 2018; Schroth, 2019; Tranta et al., 2021).

Research has emphasized how museums can design their online presence in order to increase the effectiveness of customers’ fruition, from the point of view of more immersive visits, of the coproduction of knowledge and narratives, and in terms of stimulating a visit, or a repeat visit, online or physically (Burton et al., 2009; Styliani et al., 2009; Sylaiou et al., 2010; Pallud & Straub, 2014; Mohd et al., 2018). This is link to a potentially more proactive role of customers, through web 2.0 technologies and social media, where the museum is not anymore (fully) in control of the usage and kind of experiences its consumers make of its posts, symbols, images and knowledge pieces (Arnaboldi & Diaz Lema, 2021a, b; Ryder et al., 2021).

Similarly to the museum’s online presence rooted on websites and social media, also virtual tours and more advanced, immersive experiences may drive online visitors to a physical visit (Carrozzino & Bergamasco, 2010; Lee et al., 2020; López et al., 2010), but can also open issues on how omnichannel strategies can modify consumers’ behavior (from online to physical fruition) were also museums will have to cope with “phygital” challenges (Debono, 2021), a concern still much felt at museum management level.

Issues are related to the level and diffusion of online museum offering. Still very few museums in Italy are online with state-of-the-art offering, and use advanced digital technologies (Cicerchia & Solima, 2021; Statista, 2020), thus opening questions on how such multifaced offering may affect the cultural consumer’s behavior. Various studies have suggested that the behavioral intentions of visitors of online museums should be the object of further study (Deng et al., 2010; Hassenzahl and Tractinsky, 2006; Pallud & Straub, 2014). Museum websites have witnessed an evolution, both technologically, and from the content and purpose point of view (Gaia, et al., 2020), and their role, reason of being and challenges are a highly debated topic among practitioners. However, studies of consumer behaviour on museum websites, or of their preferences, or behavioural outcomes, are still scarce. Even more so, in the case of elusive, digital native young adults such as those belonging to Gen Z (Hume, 2011; Kluge-Pinsker & Stauffer, 2021; Manna & Palumbo, 2018; Walsh et al., 2020; Tranta, 2021).

### Cultural consumption in the COVID-19 scenario

In the context of the pandemic, some of the hanging threads in culture consumption research become gaping holes. On the one hand, due to reduced personal mobility, digital exposure increased dramatically during the pandemic, and particularly during strict lockdown periods: for instance in the UK, a 29% increase on the time spent online, and a 20% increase of people using social media were reported over the first month of lockdown (Data Reportal, 2020). This led to an increase in demand for online cultural products, as well as online entertainment in general (Data Reportal, 2020). Similar numbers have appeared in statistical data all over the western world, including in Italy where online presence grew to over 100% change in a few weeks during the first lockdown, moving the country’s population on, from being digitally underconnected (Statista, 2022). On the other hand, mobility restrictions had cultural producers see their audiences vanish from their premises, for weeks on end. State museum visitors in Italy dropped by 19 million in the three months of March to May, year on year: a 78 million Euro revenue loss (Osservatorio Innovazione Digitale nei Beni e Attività Culturali, 2021). In this scenario, by no means an Italy-specific one, governments have relied on policies supporting culture and art creation during the lockdown, as a way of supporting workers and institutions (Radermecker, 2021), as well as consumers, since consumption of cultural products was deemed a way to reduce psychological and emotional challenges experienced during the crisis.

Through the combined effects of forced space constraints and of incentives to digital consumption in many realms, both state-issued and offered by private firms, the population in different countries had (and further built over time) the occasion for unprecedented digital exposure, access and experience. Because of the increased stay-at-home-consumer pull, an evolution in the offering of digital/virtual products emerged both at private business, and at public level (online schooling, telemedicine, remote public services, etc.), which in turn prompted consumers to evolve their digital skills and creativity (Pietro, 2021). Despite such positive trends related to online expanded possibilities, scholars also emphasized the potential negative consequences connected to the online dimension of users during the pandemic, such as in the case of the Gen Z (Liu et al., 2021).

In this setting, museums and cultural spaces were under pressure as to the possibility of supporting fruition at a distance, in addition to managing physical access, when it was restored albeit with restriction. Many museums invested, in order to expand or transform their offering through digital technologies (Agostino, Arnaboldi and Lampis, 2020b; Samaroudi et al., 2020). NEMO data show that more than 10% of museums increased their online presence by 100%, and more than 40% of museums increased their online active presence by one third (NEMO, 2021). By June 2021, 80% of Italian museums had created one content for their website, and 48% and 45% respectively had provided online workshops for schools, or offered guided online tours; 27% offered podcasts, and courses and games were also categories that increased (Osservatorio Innovazione digitale nei beni e attività culturali, 2021).

Within the broad audience of museums, research focuses its attention on the segment of Gen Z. This segment has become a relevant potential group of customers in many markets, and one with a strong connection with digitalization in their consumer behavior (Agrawal, 2022; Dash et al., 2021), albeit with limited analysis in the museum realm. As scholars suggested, in fact, consumers belonging to the Gen Z are more prone to use digital technologies and social media, particularly to support social interaction among peers, enhance their purchasing processes, and support their consumption activities, than are other customers (Kushwaha, 2021; Liu et al., 2021). Moreover, recent studies are suggesting that COVID-19 had an impact on the Gen Z’s orientation and behavior in multiple directions (Becker, 2022), not necessarily only in positive terms. Nevertheless, specifically through their pro-active role towards technologies, such group of customers may become an interesting target for museums, that might have benefited from the pandemic constraints to hook those customers online, with potential long-terms consequences and habit stickiness (Broersma & Swart, 2021).

*Vis-à-vis* such a theoretical and empirical scenario, it becomes critical to further develop new knowledge on how consumers reacted to the increased digital consumption engendered by the pandemic situation, in terms of cultural behavior. Specifically, the paper aims at exploring how cultural consumption patterns have been impacted by the COVID-19, both online and in terms of orientation towards the physical dimension. It takes into account the yet not fully developed museum offering in terms of digitalization, as well as specifically Gen Z. Research does highlight how data on consumer practices are still limited, and deserves further studies (Radermecker, 2021). Consumers may have different approaches and cultural consumption behaviors depending on multiple factors (Kang, 2010). Based on their attitudes, they may have multiple interests in approaching and consuming culture. Based on the above-mentioned scenario, our research intends to explore cultural consumption online with specific reference to museum products, so as to uncover to what extent COVID-19 has modified online cultural consumption and behaviour in the Gen Z.

## Methodology

### Empirical design

The research was conducted geared to elicit an exploration and a better understanding of the motives, preferences, levels of satisfactions and cultural consumption patterns of young museum goers in Italy (belonging by age to Gen Z), over two different periods and context settings: (1) before the beginning of the first pandemic lockdown in Italy (start date: 03/09/2020), and (2) during the first lockdown (end date of stricter phase one: 05/04/2020; gradual phase off until 06/04/2020). Museums were allowed to open from May 18th, although only 2% of the museums did open, and most museums reopened on the 4th of June or later. Lockdown in Italy was quite strict, and over its duration people were only allowed out of their homes for a predetermined set of primary needs. Thus, during lockdown the main entertainment opportunities besides socializing with cohabitants were online and TV ones.

The study employed a quantitative methodology based on two CAWI surveys. The surveys were administered to a sample of Italian undergraduate students from the Department of Cultural Heritage of the University of Padova. The students participating in the survey were of an age group that is typically not keen on museum consumption: 18–25 years (Gen Z) (Hume, 2011). Thus, the sample is apt to providing novel and much needed understanding on this typically non-demand segment (Kluge-Pinsker & Stauffer, 2021; Tranta, Alexandri and Kyprianos, 2021).

The surveys were intended to explore the students’ online cultural consumption habits and preferences in general and with specific respect to museums, and were temporally discriminated. The first survey (pre-pandemic sample) was administered before and during the first Italian lockdown (running dates: 03/09/2020 to 04/20/2020). The questions in this first survey were geared to explore pre-pandemic consumers’ cultural consumption habits, and concerned both online and offline cultural consumption, with a focus on museum products. This survey comprised 67 questions and had an average completion time of 19 min. The second survey (during-lockdown sample) was administered when lockdown was in place, and museums were still closed (running time: 05/04/2020 to 06/04/2020). The survey’s questions investigated consumer cultural consumption practices during lockdown, and thus focused only on online cultural consumption activities. This second survey comprised 61 questions, taking an average of 18 min to complete.

Both surveys were conducted using a mix of closed and open-ended questions and were tested on a subsample of 15 students before being rolled out on the full cohort. An incentive to participation and completion was provided to students who would participate in, and complete, both surveys. The survey is thus based on self-reported data. While this kind of data can be subject to recollection bias, it is accepted in research that consumers will remember preferences quite accurately, but consumption patterns much less so (timing and frequencies, intensity) (Haugtvedt et al., 2008), the data on consumption intensity of online entertainment emerging from our sample does display consistency with other Generation Z samples on internet consumption in literature. Thus, we can consider the sample has reported quite faithfully, which is also typical of self reported data in exceptional situations, as the novelty and shock consolidates memories as exceptional, too (Haugtvedt et al., 2008).

A total of 1003 students (pre-pandemic sample = 462, average age = 23 yo; during-lockdown sample = 541, average age = 24 yo) responded with full survey completion; 150 questionnaires were not complete and were expunged from the analysis.

The variables were comparable in the two surveys and were grouped in clusters, with logical discriminants creating different completion paths for respondents. Answers were either in option, or in a 5-point Likert format, as specified in the relevant Tables in the Results section. We can describe 3 main questionnaire areas: (a) sample characteristics and general Internet usage, both in terms of general online activities, and of cultural entertainment activities (as defined by the EU Cultural and Creative Sectors and commonly adopted in extant literature); (b) online museum website consumption patterns and evaluation (Kabassi, 2016); (c) patterns of online cultural consumption for museum non-visitors (motivations, social media use, relationship between offline and online museum products consumption).

### Method of analysis

The mirrored questions in the two surveys allow for a comparison of the habits and preferences of participants in the two settings, when in-presence visits were possible (pre-pandemic), and when they were not (during lockdown), making room for considerations around consumer learning, assuaging, differences in uses of a same product depending on environmental context, as well as the role of museum products for entertainment during such a delicate period. The main goal of the study was to assess the presence of significant differences in terms of digital cultural consumption due to the pandemic restrictions that “pushed” people into spending more time on the Internet. In this regard, in order to capture the role of the amount of time spent on the Internet and thus verify if it affected the respondents’ behaviors, we decided to split each one of the two main samples (pre-pandemic and during-pandemic) into two main groups: (1) a group that spent 4 h or less online daily (low time), and (2) a group that spent more than 4 h online daily (high time). As we expected, before the pandemic, most of students (respondents) spent less than 4 h on Internet (n = 323; 72.0% of the pre-pandemic sample) and had low Giga endowments, whereas during lockdown students had to increase the Giga (particularly, to unlimited Giga) to cater for a higher number of hours spent on the Internet (n = 429; 79.3% of the during-pandemic sample).

However, in this way we have identified 4 main groups, which allowed us to better explore the digital cultural consumption of Gen Z, and whether they modified their habits during the pandemic. Specifically, the groups refer to: (1) students that in the pre-pandemic period spent online 4 or less hours per day (n = 305; 66.0% of the sub-sample pre-pandemic, (2) students that in the pre-pandemic period spent online more than 4 h per day (n = 157; 34.0% of the sub-sample pre-pandemic), (3) students that during the lockdown spent online 4 or less hours (n = 112; 20.7% of the sub-sample during-pandemic), and (4) students that during the lockdown spent online more than 4 h (n = 429; 79.3% of the sub-sample during-pandemic). As main method of analysis, to reach the research purposes, the resulting groups were statistically analyzed considering the time spent online and comparing the two periods, pre-pandemic and during-lockdown, to assess differences in terms of digital cultural behaviors, with respect to the generic digital activities and both the online museum visiting and non-visiting. Moreover, to check if the time spent online had a relevance in the students’ digital behaviors, we compared within each sample (pre- pandemic and during-lockdown) the two time’s groups (low time spent online and high time spent online). In doing so, we performed ANOVA and Chi-square tests using SPSS 28.0.

## Results

### General digital and cultural consumption pre- and during lockdown

The lockdown imposed by the Italian government during the first stage of the COVID-19 pandemic in the spring of 2020 forced people to spend more time on the Internet, both for work and study purposes, and for leisure and other activities. Table 1 reports the purposes behind Internet time. Specifically, as expected, during the lockdown, independently from the hours spent online (thus for both groups referred to as 4 or less, and more than 4 h online) students spent, respect to the pre-pandemic period, more time for university activities (i.e., lectures and videoconferencing) because of the distance-learning activities, but also for physical exercise.

**Table 1:** Activities performed online

	Lower time spent on Internet			Higher time spent on Internet		
	Pre-pandemic	During lockdown	Sig	Pre-pandemic	During lockdown	Sig
Studying/Work	3.45	**4.08**	.000	3.68	**4.41**	.000
Physical exercise	1.87	**2.80**	.000	1.86	**2.93**	.000
Cultural entertainment	2.94	3.09	.218	3.13	3.14	.916
Social networks	**4.04**	3.76	.021	4.28	4.17	.226
Playing games online	1.86	2.02	.197	2.11	2.21	.443
Videoconferences	2.66	**3.69**	.000	2.63	**3.89**	.000
*N*	305	112		157	429	

ANOVA analysis. Average values (5-points Likert scale). Values in bold are the higher values of the statistically significant differences

It is interesting to note that students spending less time online in the pre-pandemic period significantly employed higher time on social networks with respect to the during-lockdown period, and that in both periods the cultural entertainment is positioned in the middle, in terms of relevance.

Focusing on digital cultural and arts experiences (included museum and other virtual tours), Table 2 gives a representation of what survey participants consider as “cultural entertainment” and whether this perception changed over the pre-pandemic and lockdown periods.

**Table 2:** Digital cultural entertainment (not include study-related activities)

	Lower time spent on Internet			Higher time spent on Internet		
	Pre-pandemic	During lockdown	Sig	Pre-pandemic	During lockdown	Sig
Listen to music	**72.1%**	56.3%	.002	**77.1%**	59.9%	.000
Create and publish music contents	5.2%	***9.8%***	.092	10.8%	9.8%	.712
Reading books, newspapers, magazines	58.7%	**70.5%**	.027	58.6%	**73.9%**	.000
Publish articles and other texts	5.2%	**13.4%**	.005	4.5%	**11.7%**	.009
Watch theatre shows	19.7%	25.0%	.237	12.1%	**33.1%**	.000
Perform live-videos (ex. Facebook Live)	1.3%	**4.5%**	.050	0.6%	**4.2%**	.031
Watch short- or long films/videos	54.8%	58.0%	.550	57.3%	60.4%	.506
Making short/full-length videos or docu-videos	5.9%	***10.7%***	.092	13.4%	9.1%	.130
Attending webinars or videoconferences	9.5%	**39.3%**	.000	10.8%	**38.7%**	.000
Visiting websites of cultural organizations (museum, private collections, UNESCO sites, etc.)	52.1%	57.1%	.363	54.1%	**67.1%**	.000
Visiting websites of museum or church	42.3%	**62.5%**	.000	47.1%	**59.2%**	.009
Following a museum or city virtual tour	28.9%	**54.5%**	.000	26.1%	**56.6%**	.000
Visiting or managing a blog	15.4%	12.2%	.456	9.6%	***14.9%***	.092
Publishing artistic photos/pictures	**35.1%**	16.1%	.000	31.8%	27.5%	.303
*N*	305	112		157	429	

Chi-square analysis. Values in bold are the higher values of the statistically significant differences with a *p*-value lower than 0.05. Values in bold and italic are the higher values of the (marginally) statistically significant differences with a *p*-value lower than 0.10

Before the pandemic, our respondents considered cultural entertainment mainly as listening to music online and this is not linked to the amount of time spent online; indeed, this result is significantly higher with respect to the lockdown period both for the group that spent less, and more time online. Moreover, in the pre-pandemic period, a statistically significant higher percentage of respondents, which spent low time online, considered publishing of artistic photos/pictures as cultural entertainment.

On the other hand, during lockdown, the meaning of “cultural entertainment”, for our sample shifted. The results referred to the two different time groups are quite similar. Particularly, for what concerns the pre-pandemic period, during lockdown the digital cultural entertainment changed significantly. The most relevant significant differences concern the much higher percentage considering cultural entertainment as reading books, newspapers and magazines online, visiting web-profiles (websites or social networks pages) of cultural organizations (museum, private collections, UNESCO sites, etc.), visiting the websites of museums or churches, and going on a museum or city virtual tour, as well as attending webinars by cultural organizations, watching theatre shows and publishing articles or contents, and both conducting, and attending lives/webinars.

In addition, other interesting differences concern social networks and specifically, the following of a museum, especially for the respondents that spent more time online. Pre-lockdown, 46.5% of respondents followed at least one museum’s profile on social media, but this data jumps to 57.1% during lockdown, with *p* < 0.050. Conversely, there are no differences in terms of social media page visit frequency, which remains stable for the majority of respondents.

Checking the role of time spent online in the two different periods assessed, before and during the pandemic, it is interesting to note that with respect to proactive/creative digital cultural activities there are only few significantly differences. Before and during the pandemic, spending more time online enabled students to increase (pre-pandemic) the creation and publishing of creative contents (videos and music) and (during lockdown) to increase the publishing of artistic photos/pictures.

### The museum visiting online experience

According to the main research goal, which concerns the exploration of possible changes occurred during the lockdown with respect to the digital experiences regarding the online visiting of a museum, in this section we focus on those students that have experienced both in the pre-pandemic, and during lockdown the visiting of a museum website. In this regard, the main difference between the pre and lockdown groups concerns the chi-square analysis on whether the respondent has visited a museum website during the period or not. Data reveal that during lockdown the % of students that visited a museum website increased significantly (247 students, 45.7% of the lockdown group vs. 132 students, 28.6% of the pre-pandemic group; *p* < 0.001). The difference between pre-pandemic and during lockdown is higher for the group that spent more time on Internet (46.9% during lockdown *vs* 27.4% pre-pandemic, *p* < 0.001) than for the group that spent less time on Internet (41.1% during lockdown *vs* 29.2% pre-pandemic, *p* < 0.05). This is particularly striking, if we consider that students responding to the survey were not requested to visit online museums because of coursework, but they spontaneously resorted to this activity in their leisure time.

The rest of the analysis focuses on extant differences in visiting a museum website in the two periods and with respect to the amount of time spent online (4 or less hours/more than 4 h). Table 3 highlights the activities performed during the visiting of a museum website. In the pre-pandemic period, students mainly performed, in a significantly different way with respect to lockdown, basic activities such as looking for information, downloading images and playing games.

**Table 3:** Activities performed during the visiting online of a museum

	Lower time spent on Internet			Higher time spent on Internet		
	Pre-pandemic	During lockdown	Sig	Pre-pandemic	During lockdown	Sig
Looking for information	**71.9%**	32.6%	.000	**69.8%**	42.3%	.001
Perusing & reading about collection	46.1%	**67.4%**	.019	55.8%	63.2%	.366
Taking a virtual tour	37.1%	**69.6%**	.000	34.9%	**70.1%**	.000
Playing games	**16.9%**	4.3%	.038	2.3%	4.1%	.601
Download images	***25.8%***	13.0%	.086	***30.2%***	17.4%	.055
Watching videos	40.4%	41.3%	.924	27.9%	**47.8%**	.017
Attending webinar	9.0%	15.2%	275	4.7%	**11.9%**	.160
*N*	89	46		43	201	

Chi-square analysis. Values in bold are the higher values of the statistically significant differences with a *p*-value lower than 0.05. Values in bold and italic are the higher values of the (marginally) statistically significant differences with a *p*-value lower than 0.10

Instead, during lockdown, with respect to the pre-pandemic period and independently from the amount of time spent online, students performed more engaging activities, such as taking a virtual tour and watching museum videos and art collections.

Moreover, respondents’ behavior in exploring the collection during the visit online changes (evolves) from before, and during lockdown. Indeed, as shown in Table 4, in the pre-pandemic period, students visiting the museum online performed the exploration of the museum collection by mainly perusing the images (data significantly higher with respect to the lockdown period) and in the case of more time spent online they preferred to follow a logical route rather than following a virtual digital tour, with respect to students that during lockdown spent less time online. During lockdown, however, a significantly higher percentage of students, with respect to the pre-pandemic period, showed more mature behaviors, as they explored the collections by reading the descriptions of the images, as well as followed a guided virtual tour during the online visit in both time conditions spent online, thus seeking a more rich and enriching experience, a finding that might please museum managers.

**Table 4:** Exploration of the collection over a museum website visit

	Lower time spent on Internet			Higher time spent on Internet		
	Pre-pandemic	During lockdown	Sig	Pre-pandemic	During lockdown	Sig
Browsing	**59.2%**	8.7%	.000	**35.0%**	13.9%	.000
Reading the description of the collection	2.6%	**15.2%**	.000	2.5%	**24.4%**	.000
Following a logical digital route	22.4%	**37.0%**	.000	**37.5%**	22.9%	.000
Following a guided virtual digital tour	15.8%	**39.1%**	.000	25.0%	**38.8%**	.000
*N*	89	46		43	201	

Chi-square analysis. Values in bold are the higher values of the statistically significant differences with a *p*-value lower than 0.05

Another area providing interesting differences concerns the experience attributes most appreciated during the museum website visit. These results are displayed in Table 5. Also in this case, the analysis highlights a more mature experience during lockdown, especially when students spent more time online, as they significantly appreciated both the learning and involvement aspects of the digital experience, but also appreciated the opportunity to have more time to watch and enjoy the art collections without being distracted as may happen during the physical visit, but they also appreciated the specificity of a digital experience that differs from the offline one.

**Table 5:** Experience attributes appreciated visiting a museum online

	Lower time spent on Internet			Higher time spent on Internet		
	Pre-pandemic	During lockdown	Sig	Pre-pandemic	During lockdown	Sig
Learning experience	3.88	3.97	.632	3.97	**4.35**	.025
Aesthetic experience	3.59	3.50	.667	3.90	4.05	.444
Involving experience	3.39	3.56	.356	3.55	***3.89***	.073
Various activities experienced	3.00	2.91	.715	3.28	3.27	.970
Logical path guiding the visiting	3.34	**3.91**	.013	3.24	**3.70**	.022
Differences with physical visit	2.97	3.09	.562	2.83	**3.46**	.007
More time to enjoy the collection	3.00	**3.56**	.033	2.83	**3.83**	.000
Enjoying the collection without being distracted	3.36	3.69	.252	3.55	***4.00***	.082
*N*	59	32		29	171	

ANOVA analysis. Average values (5-points Likert scale). Values in bold are the higher values of the statistically significant differences with a *p*-value lower than 0.05. Values in bold and italic are the higher values of the (marginally) statistically significant differences with a *p*-value lower than 0.10. Respondents answered to this question only if they chose the positive options (positive or very-positive) to the question “How do you evaluate the online visit of a museum?”

However, despite the differences in pre- and lockdown periods regarding the appreciation of attributes of the online experience between pre-pandemic and lockdown, no difference emerges in terms of overall liking of the online experience: most respondents of both surveys (70.4% lockdown and 66.7% pre-pandemic) considered the online experience as very positive, independently from the time spent online. Moreover, there emerge no differences with respect to the influence that the online visiting may have on the physical visiting of a museum. Here too, for the majority of respondents in both surveys (61.1% lockdown and 56.1% pre-pandemic), the visit online positively affects the physical visiting of a museum. In turn, the in-presence visit is widely preferred to the online visiting (97.0% of respondents pre, and 97.2% during lockdown preferred the physical visit).

It is interesting to note that analyzing the effect of time spent online with respect to the activities performed and the exploration of the collection while visiting the museum website in the pre-pandemic and during lockdown, there are no differences. Instead, with respect to the attributes, during lockdown respondents that spent more time online highly appreciated both the learning (4.35 during lockdown vs 3.97 pre-pandemic, *p* < 0.05) and aesthetic (4.05 during lockdown vs 3.50 pre-pandemic, *p* < 0.01) experiences.

Finally, taking into consideration the motivations that pushed respondents to visit a museum website and have a cultural digital experience, as shown in Table 6, it is interesting to note that there are significant differences between the pre-pandemic period and the lockdown, mainly when students were heavy internet users. Indeed, during lockdown, and with respect to the pre-pandemic period, respondents chose to visit a museum website and have a digital experience because they found it as interesting as the real experience, in addition to being involved in the digital activities offered by the museum. As already stated with respect to the relevance of digital and physical experiences, the digital experience is equally perceived by both groups as a useful “training" for the real experience.

**Table 6:** Motivations of visiting a museum website

	Lower time spent on Internet			Higher time spent on Internet		
	Pre-pandemic	During lockdown	Sig	Pre-pandemic	During lockdown	Sig
I found it interesting doing online what I like to do offline	44.9%	56.5%	.202	32.6%	**49.3%**	.046
To be ready for the offline visiting	40.4%	54.3%	.124	53.5%	52.7%	.929
To do the cultural activities offered by the museum	5.6%	**23.9%**	.002	7.0%	**28.9%**	.003
Because it is interesting	39.3%	45.7%	.479	32.6%	**55.2%**	.007
*N*	89	46		43	201	

Chi-square analysis. Values in bold are the higher values of the statistically significant differences with a *p*-value lower than 0.05

### The museum website non-visitors

After having explored the main features of visiting a museum website and thus of a museum digital experience by Gen Z, the last step of our analysis concerned the assessment of the online activities performed by those participants that had not visited an online museum in the surveyed period, and of the reasons why. In this case, too, we compared the two periods by taking into consideration the different amounts of time spent online: low internet time (4 or less hours) and high (more than 4 h). Among the various activities performed online by such respondents, as shown in Table 7, significant differences concern virtual tours and cultural webinars. During lockdown heavy internet users in our sample show significantly higher percentage values for watching video documentaries, for naturalistic sites’ virtual tours, and for attending cultural webinars, independently from the time spent online, suggesting a more sophisticated kind of cultural consumption for the lockdown period. Instead, as already mentioned above, in the pre-pandemic period, and with respect to lockdown, a higher percentage of students preferred to spend their (lower) time online listening music.

**Table 7:** Cultural entertainment of digital museum non-visitors

	Lower time spent on Internet			Higher time spent on Internet		
	Pre-pandemic	During lockdown	Sig	Pre-pandemic	During lockdown	Sig
Listen to music	**88.0%**	75.8%	.015	85.1%	89.9%	.191
Create and publish music contents	6.9%	6.1%	.802	9.6%	5.7%	.178
Watching movies	81.5%	81.8%	.951	86.8%	86.4%	.911
Watching videos documentaries	72.2%	71.2%	.873	65.8%	***75.0%***	.074
Producing video contents	8.3%	4.5%	.305	10.5%	8.3%	.505
Reading books / articles	45.8%	37.9%	.255	41.2%	45.6%	.441
Writing-up books / articles	8.8%	3.0%	.118	5.3%	5.3%	1.000
Participate to city virtual tours	6.5%	12.1%	.135	13.2%	18.0%	.256
Participate to nature places virtual tours	7.4%	**16.7%**	.025	10.5%	***18.4%***	.059
Attending courses and webinars	7.9%	***15.2%***	.079	11.4%	***19.3%***	.065
*N*	216	66		114	228	

Chi-square analysis. Values in bold are the higher values of the statistically significant differences with a *p*-value lower than 0.05. Values in bold and italic are the higher values of the (marginally) statistically significant differences with a *p*-value lower than 0.10

As for the reasons why these respondents had never visited a museum website, as shown in Table 8, the most relevant and significant differences, which are similar for both low and high time spent online, concern the higher percentage of respondents that, during lockdown, stated they did not have time for the visiting, even if they were shut inside their homes, and a significantly higher percentage of students that in the pre-pandemic period never thought about the possibility or opportunity to visit online a museum through the website. Almost the totality of respondents prefer the visit in presence. In this sense, part of non-demand might be stimulated by better communication and increased experience of digitalization opportunities, even for Post-Millenials.

**Table 8:** Motivations of non-visiting online a museum

	Lower time spent on Internet			Higher time spent on Internet		
	Pre-pandemic	During lockdown	Sig	Pre-pandemic	During lockdown	Sig
Not interested in the museum experience	1.4%	1.5%	.939	0.9%	3.9%	.112
Preference for offline experience	94.9%	89.4%	.109	89.5%	88.2%	.718
Lack of time	6.0%	**15.2%**	.018	13.2%	**22.4%**	.042
I never thought about this possibility	**39.4%**	22.7%	.013	**50.9%**	21.9%	.000
Preference for other activities	31.0%	33.3%	.723	36.0%	35.5%	.936
*N*	216	66		114	228	

Chi-square analysis. Values in bold are the higher values of the statistically significant differences with a *p*-value lower than 0.05

## Discussion

COVID-19 induced a digital acceleration at both supply and demand level across all industries. Museums were no exception and engaged in digital transformation: the online–onsite (im)balance resulting from lockdown and social distancing saw physical visits grind to a halt, leaving museums able to provide their products only online, and visitors stranded at home. Our explorative analysis points out that during the lockdown experienced in Italy in the spring of 2020, consumption patterns of a sample of Gen Z postmillennial museum goers, a typically elusive segment for what concerns museum demand, changed and become more mature, with a higher involvement.

The kind of activities performed online during lockdown only see a marginally significant increase in online cultural consumption, as it is the primary activities that cannot be performed in person, that need to grow the more (going to university, physical movement, connecting). However, this does not mean that cultural consumption profiles were unscathed. In fact, what changes is the very perception of what online cultural entertainment really is. If before lockdown cultural entertainment online was focused on listening to music and posting pictures, during lockdown it becomes more varied, and encompasses more complex activities such as reading, visiting cultural organizations’ websites (+ 16%), going on heritage virtual tours (+ 28.3%) and this is not linked to the amount of time spent online. Indeed, in this sense, in the same period, pre-pandemic or during lockdown, the samples of internet usage levels have not shown very significant differences. The concept of cultural entertainment gradually shifts towards more and more proactive activities. One first result of our analysis is thus that consumers appear to have opened up to a much wider range of online cultural products. These products are also more immersive and require more input on the part of the consumer (from listening to music to navigating a website, to directing a virtual tour, to producing content). These are all experiences inducing learning, and possibly a change perception of what can be achieved online.

**Fig. 1:**
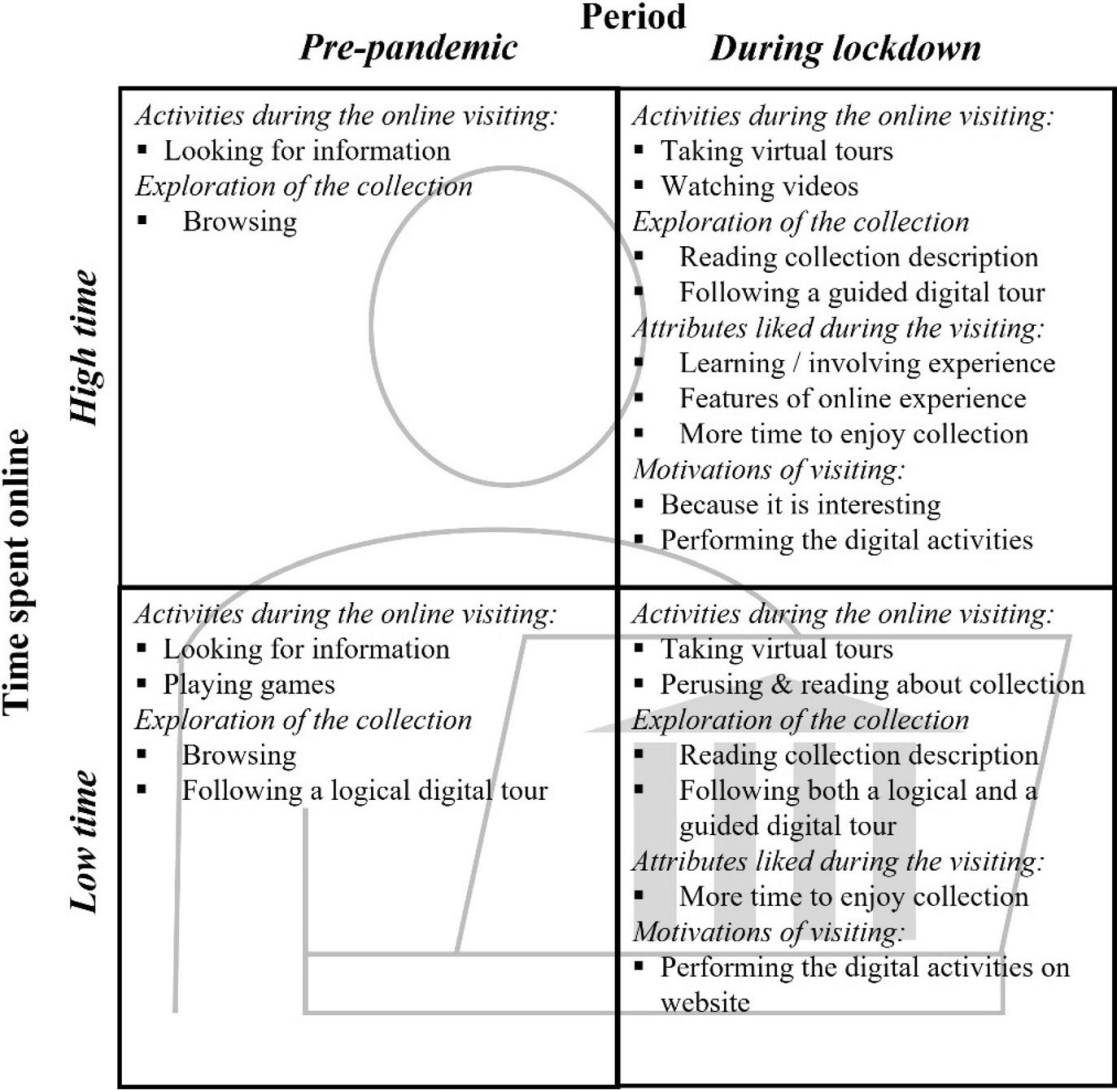
Main differences of museum online visiting in the two timeframes. Source: Authors’ elaboration

For what concerns museums more specifically, our data show that lockdown marked a significant increase in demand for online museum website visits, which totally were up 17.1%. However, these website visits were perceived as “very positive” in both periods and with no significant difference between periods. It was the behaviors of respondents during the navigation, that were different in the two timeframes. Figure 1 shows the main significant differences between the two analyzed periods, taking into consideration the two levels of time spent online, low time (4 or less hours) and high time (more than 4 h), and with respect to the activities performed during the online visits, the exploration of collections views, the attributes liked during the visiting and the motivations of visiting a museum website. Mirroring and reinforcing the result on the lockdown-ensued development of the concept of online cultural entertainment discussed above, respondents chose more advanced, engaging and input-requiring activities during lockdown, than they did before. If searching for info understandably drops, there are sharp increases in some of the most co-production prone activities, such as watching the collections, taking a virtual tour and watching videos. This piece of data is also related to the significant change in the way the activities are performed. From merely browsing the website looking at the pictures, consumers shift to now actually reading the contents and engaging in virtual tours in guided (content enhanced) mode, rather than taking the plain tour. Thus, we can say that lockdown had an impact also at a more micro level, as it not only modified the preferred activities to perform on the website, but also the way they were performed. This is a relevant finding for operators, as it points to specific avenues for museum website activity management and consumer analysis, and a relevant success criteria to attend to in museum websites (Kabassi, 2020). It is also a relevant finding for literature, as the question of both whether it is at all possible, as well as how, to spark an interest in young adults, aka making them active, rather than passive, museum consumers, is a poignant and long-standing one (Batat, 2020; Hume, 2011), and one that deserves to be unpacked.

Data on experience attributes’ liking rates also undergo a general, significant increase. During lockdown, Gen Z appreciated the possibility to enjoy the collection, the specific features of the online visiting, as well as the learning and involvement aspects of the digital experience. This is not surprising. It is a result that typically follows from consumers of a cultural product engaging more with the product itself: in cultural markets, as the levels of consumer engagement and coproduction rise, so does customer satisfaction (García-Madariaga et al., 2017). Moreover, it is also interesting to note the higher interest for digital consumption expressed during the lockdown as a motivation of the online visiting.

If lockdown stimulated the perception of a wider range of online cultural consumption possibilities, as well as more specifically the change and scope of museum website fruition, there are implications in terms of multi-channel strategies (cannibalization vs. integration between online and physical presence). If some literature findings might mildly point in this direction (García-Madariaga et al., 2017), our data show the contrary: not only a stable, overwhelming majority of respondents in both time frames prefer the physical visit to the website visit (97% and 97.2%, respectively, pre and during lockdown), but visiting the museum website positively influences users’ intentions to visit the museum in presence in the future (56.1% pre and 61.1% in lockdown). This finding is consistent with extant research not finding support for the hypothesis often discussed on the field of a supposed website cannibalization of the museum, a finding mirroring existing research both on museum website-museum cannibalization (Marty, 2007), and on other cultural productions with complementary support, such as magazines with companion websites (Kaiser & Kongsted, 2007).Finally, our research shows that the most quoted reason for not having visited a museum website in the pre-pandemic period was the lack of knowledge about the possibility of doing so. In this sense, it is necessary to improve the communication illustrating and promoting the possibilities offered by an online museum offering, to reach those consumers that are still unaware.

## Conclusions

The paper provides an original contribution in the explorative analysis of cultural consumption in the Generation Z, with specific reference to the fruition of museums’ offerings before and during the pandemic, by considering also the role of time spent online in these scenarios and utilizing the lockdown’s exceptional time as an observation lab of the effects of online hyper exposure on cultural and museum online consumption and appreciation in digital natives.

On the one hand, our study further enriches the debate on the implications of the COVID-19 lockdown for consumer habits, particularly for museums visitors (and non-visitors). This research suggests that there was an evolution in consumer behavior mainly due to consumers’ new conditions and approaches to the digital experience. On the other hand, our results point out a learning process occurred during the lockdown that may have durable implications in the future, especially in terms of more mature digital experiences, calling for cultural institutions to step up. Our research pointed out the positive, pro-museum behavior of our Gen Z sample, who reinforced their interests towards cultural consumptions in general, and on museums’ offering more specifically, during the pandemic. Our study advances research on how a specific, yet not fully observed, museum audience group approaches cultural fruition digitally. While one may expect that Gen Z rely on digital tools to support and guide their experience online, this does not mean necessarily that such attention also involves arts and culture. Our study points out that the pandemic has modified their attention towards cultural consumptions, also in terms of a more pro-active role, with changes in the behaviors that could persist over time and that do not depend on the amount of time spent online.

From a theory point of view, the study also shows the relevance of integrating the online experience in traditional museum offering. Extant literature points to the relevance of a digital environment for cultural consumption (Palumbo, 2022; Raimo et al., 2021), as it may act as a facilitator for the physical, in presence experience, which especially for the cultural and art consumption, our study finds to be preferred to the online one. We can thus confirm Manna and Palumbo’s museum visitors’ results (2018), validating them from a more exquisitely consumer point of view, in that digital and online experiences can be fundamental to provide consumers (in our case Gen Z consumers) with a more immersive and deep experience. Providing an engaging digital experience may not only improve the digital visit, but also overall consumer satisfaction, including more attention towards the physical visit. One interesting consideration is, whether such behavior may show habit stickiness. Although research on consumer learning and habits, particularly online habits, tells us that online consumption is a habit that sticks, and particularly when developed in a crisis setting (Broersma & Swart, 2021), this is a relevant issue for further research. Operators and researchers alike should be aware of the changes occurred in the perception of cultural entertainment, and focus on sustaining the learning, so that it does not get unlearned (operators). Researchers could delve into exploring such changes’ persistency and extent in other consumer cohorts, too. Operators will also benefit from our findings in this sense, as Post-Millennials are typically a non-demand segment: our study gives indication of the relevant consumption items and considerations for a very difficult segment to serve and interest enough into museums, and to even simply do research on.

Moreover, from practitioners’ point of view, museums should plan a multi-channel strategy based on the integration between online and offline experiences. They should provide a digital experience (website design) allowing consumers to increase their engagement through different activities (Pallaud and Straub, 2014), thus improving both the hedonic experience (enjoying the cultural and art collections) as well as the utilitarian experience (learning about how get the best from cultural consumption). In so doing, museums should think about strategies that push for an integrated consumption of online and offline experiences (Trunfio et al., 2020). In this sense, museums should also focus on an effective communication strategy, also thorough the use of social networks (Ryder et al., 2021), pushing the online experience, too.

As our study focuses on a specific consumer segment, such limitation could be hints for additional research considering other countries and customers’ profiles (i.e. based on age). Another limitation of our study is related to the analysis of customer online behavior directly focusing on consumers and not including also data provided by museums and how their offering has been used by customers during the lockdown. Future research may seek to match demand behavior with the museum’s online offering (supply), also addressing the customer’s behavior during the post-lockdown period, to provide additional elements to the analysis of the learning dynamics.
